# Environmental Factors and Their Threshold Affecting the Survival of Five Aquatic Animal Viruses in Different Animal Cells

**DOI:** 10.3390/v14112546

**Published:** 2022-11-17

**Authors:** Zi-Hao Wang, Fei Ke, Jian-Fang Gui, Qi-Ya Zhang

**Affiliations:** 1 State Key Laboratory of Freshwater Ecology and Biotechnology, Institute of Hydrobiology, Chinese Academy of Sciences, Wuhan 430072, China; 2 College of Modern Agriculture Sciences, University of Chinese Academy of Sciences, Beijing 100049, China

**Keywords:** aquatic animal viruses, environmental factors, UV, temperature, pH, drying, titers, mammalian cell

## Abstract

Aquatic animal viruses infect and transmit in aquatic environments, causing serious harm to the aquaculture industry and a variety of wild aquatic animals. How are they affected by environmental factors and do they represent potential threat to mammalian heath or not? Here, the effects of environmental factors (ultraviolet radiation (UV), temperature, pH, and drying) and their threshold on five epidemic aquatic animal viruses infecting amphibians and bony fish, including *Rana grylio* virus (RGV), *Andrias davidianus* ranavirus (ADRV), Grass carp reovirus (GCRV), *Paralichthys olivaceus* rhabdovirus (PORV), and *Scophthalmus maximus* rhabdovirus (SMRV), were measured and compared in a fish cell line. The examination of virus titers after different treatment in fish cells showed that the two iridoviruses, RGV and ADRV, had a higher tolerance to all of the environmental factors, such as they only had a decay rate of 22–36% when incubated at 37 °C for 7 days. However, the rhabdovirus SMRV was sensitive to all of the factors, with a decay rate of more than 80% in most of the treatments; even a complete inactivation (100%) can be observed after drying treatment. To address the potential threat to mammals, infectivity and limitation factors of the five viruses in Baby hamster kidney fibroblast cells (BHK-21) were tested, which showed that three of the five viruses can replicate at a low temperature, but a high temperature strongly inhibited their infection and none of them could replicate at 37 °C. This study clarified the sensitivity or tolerance of several different types of aquatic animal viruses to the main environmental factors in the aquatic environment and proved that the viruses cannot replicate in mammalian cells at normal physiological temperature.

## 1. Introduction

Viruses are major aquatic microorganism players which can cause devastating or transboundary diseases. In recent years, the rapid development of aquaculture industry has made outstanding contributions to the protection of global food nutrition and safety [1,2]. However, viral diseases have seriously threatened the production and quality of aquatic products [3,4]. There are a wide variety and a large number of viruses in the aquatic environment and they are even transmitted cross-species [5,6,7], such as members of the families *Iridoviridae*, *Rhabdoviridae*, and *Reoviridae*. These viruses include enveloped (*Iridoviridae* and *Rhabdoviridae*) or non-enveloped (*Reoviridae*) capsids, and double-stranded DNA (dsDNA), double-stranded RNA (dsRNA) or single-stranded RNA (ssRNA) genomes, which could infect specific host or non-natural host, potentially threatening aquacultural and wild aquatic animals [8]. So far, information about isolation, taxonomy, genome sequence of several aquatic animal viruses has been reported [9,10].

*Rana grylio* virus (RGV) and *Andrias davidianus* ranavirus (ADRV) are ranaviruses isolated from diseased pig frog *R. grylio* (anura amphibian) and Chinese giant salamander *A. davidianus* (urodele amphibian), respectively. They are enveloped large dsDNA viruses within the genus *Ranavirus* in the family *Iridoviridae* [11,12,13]. Due to its interspecies infection character, several members in the genus *Ranavirus* represent great threats to the aquaculture industry and wild animal populations [14,15,16]. Grass carp reovirus (GCRV) is aquareovirus isolated from diseased grass carp (*Ctenopharyngodon idellus*) with a genome composed of 11 segments of dsRNA [17]. It is a non-enveloped virus within the family *Reoviridae*, and mainly infects yearling and fingerling grass carp, and other cyprinids. Grass carp aquaculture accounts for a large share of freshwater aquaculture in China, and, therefore, outbreaks of grass carp hemorrhagic disease have caused great damage to the aquaculture industry every year [18]. *Paralichthys olivaceus* rhabdovirus (PORV) and *Scophthalmus maximus* rhabdovirus (SMRV) are rhabdoviruses isolated from diseased flounder *Paralichthys olivaceus* and turbot *Scophthalmus maximus*, respectively. They are enveloped viruses with a genome consisting of a non-segmented negative-sense ssRNA within the family *Rhabdoviridae* [19,20]. The rhabdovirus can cause severe hemorrhagic septicemia in both freshwater and marine fishes, therefore needs to be given attention [21]. The five viruses all can replicate in fish cell lines from one species. For example, they all infected well in *Epithelioma papulosum cyprinid* (EPC) cells, which is a commonly used cell line in aquaculture research [12,13,19,22,23].

Considering these viruses exist in aquatic environment, the survival of the viruses are affected by different environmental factors. Which are the major environmental factors affecting the stability of the viruses? Previously, discrepant virus abundance and activities were observed in various strata of the sea along with different physicochemical factors [24], and virus prevalence was also observed to be correlated with environmental factors [25]. For aquatic animal viruses, an investigation of the survival times of several fish viruses in the natural water bodies [26], the effect of water temperature on the occurrence of shrimp white spot virus disease [27], and the persistence of a variety forms of ranaviruses in the aquatic environment [28] have been reported. However, until now, a comparative analysis of the effects of important environmental factors and their effect degrees on different aquatic animal viruses are still lacking.

In addition, some aquatic animal viruses have been reported to possess interspecies infection ability [16,21], which has given rise to some concerns that aquatic animal viruses may threaten human health in the context of the current pandemic [29]. Whether aquatic animal virus can infect mammalian cells or if there are some limitations for their infection in mammalian cells remains unknown. 

In the present study, the five aquatic animal viruses (RGV, ADRV, GCRV, PORV, and SMRV) were selected as representatives, and used to measure and compare the effect of environmental factors such as UV radiation, temperature, pH, and drying stresses on the virus titers; we also assessed their infection ability in a cultured mammalian cell line.

## 2. Materials and Methods

### 2.1. Cell Lines and Culture

EPC cells were maintained in Medium 199 supplemented with 10% fetal bovine serum (FBS, TianHang, Hangzhou, China) at 25 °C [30]. When used for stock virus preparation, cells were cultured in Medium 199 supplemented with 5% FBS in T75 cell culture flasks. *Siniperca chuatsi* skin cell line (SCSC) that was established in our lab was maintained in L-15 medium with 10% FBS at 25 °C and was used in TCID_50_ assay to determine virus titers [31]. Baby hamster kidney fibroblast cells (BHK-21) were cultured in Dulbecco’s modified Eagle’s medium (DMEM, Gibco, New York, China) supplemented with 10% FBS at 37 °C in 5% CO_2_ [32].

### 2.2. Preparation of Stock Viruses and Titration

RGV, ADRV, GCRV, PORV, and SMRV were previously stored at −80 °C in our laboratory [12,13,18,19,20]. All of them were repropagated by EPC cells. In detail, 100 μL of each virus suspensions were inoculated into a T75 flask pre-inoculated with EPC cells, which were incubated at 25 °C except PORV at 15 °C and harvested when cytopathic effect (CPE) reached about 80~90% of the cell monolayer. The harvested cells were subjected to three freeze–thaw cycles. The viruses were aliquoted and stored at −20 °C until use.

For virus titration, 96-well plates containing 100 μL of confluent SCSC cells were infected with 100 μL of viruses with 10-fold serial dilutions (10^−1^ to 10^−8^). Titrations were carried out in triplicate, and subsequent experiments were conducted under this condition. Infected cells were incubated for 7 days at 25 °C except PORV at 15 °C, and the CPE was recorded. TCID_50_ was determined according to the previous study [33] to represent the infectivity of each virus.

### 2.3. UV Treatment

To study the effects of UV on the viruses, virus suspensions of RGV, ADRV, GCRV, PORV, and SMRV were added to sterile 6-well plates, then were treated with UV (5 × 10 W germicidal lamp that emitted monochromatic UV at 254 nm, 5 cm from the lamp, UV exposure energy density 0.3 J/min) at room temperature. Initial virus suspensions were titrated and used as 0 h data. After UV exposure for different times (5, 10, 20, 40 and 60 min) (the energy was 1.5, 3, 6, 12 and 24 J, respectively), each virus was collected for titration using the TCID_50_ assay. To compare the sensitivity of different viruses to the treatment, the decay rate was used to show the decrease degree of the virus titer, which is calculated using the equation: decay rate = (lg*iTCID_50_*-lg*fTCID_50_*)/lg*iTCID50*. *iTCID_50_* indicates the initial titer of each virus, and *fTCID_50_* indicates the final titer obtained after different treatment.

### 2.4. Incubation at Different Temperatures

To investigate the effect of incubation at different temperatures on the viruses, virus suspensions of RGV, ADRV, GCRV, PORV, and SMRV were added into 1.5 mL EP tube and placed at 4, 15, 25, and 37 °C, respectively. Then, 150 μL of each virus suspensions were taken for titration as described above after incubating for 1, 3, and 7 days. Before incubating at different temperatures, virus suspensions were titrated and used as 0 days data.

### 2.5. pH Treatment

To clarify the effect of various pH on the viruses, pH values of PBS were adjusted to 3, 5, 7, 9, and 11 using 12 M HCl and 6 M NaOH, and then autoclaved for decontamination prior to provide different pH environments for the viruses. The virus suspensions were mixed with 10 times the volume of PBS at different pH, respectively, and incubated at 4 °C. A total of 150 μL of each virus suspensions were taken after incubating for 0, 1, 3, and 7 days, firstly neutralized by 1 M HEPES solution (Procell, Wuhan, China) to pH 7, and then used for titration as described above.

### 2.6. Drying Treatment

In order to probe into the effect of drying on aquatic animal virus infectivity, 150 μL of each virus suspension was placed in individual wells of a sterile 24-well plates and allowed to dry at room temperature (25 °C) and a relative humidity (RH) of 70–80%. The lids of plates were half-opened to keep air circulating with the environmental chamber. The environmental conditions were produced in a biochemical incubator. During the evaporation period, the volume of residual virus suspension was measured and recorded at the time point (0, 0.25, 0.5, and 1d), and then fresh medium was added to 1500 μL and used for titration after resuspension. Once it entered the drying phase, at each time point (1,5, 2.5, 4.5, and 7 d), samples were collected by 10~15 min incubation and rinsed with 1500 μL medium, and then used for titration.

### 2.7. Virus Infection of BHK-21 Cells

For determination of the sensitivity of the five viruses to BHK-21 cells, the cells were placed into 24-well plates and cultured at 37 °C to obtain a monolayer. The virus suspensions were added into each well at a dose of 100 TCID_50_, and then incubated at 28 °C for 1 h. After incubation, the virus suspensions were removed and a fresh medium was added. The infected cells were cultured at 28 °C and collected at 12 and 24 h post-infection (hpi) for virus gene expression detection.

To test the appropriate temperature for infection of the ranaviruses (RGV and ADRV) in BHK-21 cells, two recombinant viruses (ΔTK-RGV and ADRV_46R-3Flag_) that we constructed in our previous study and that expressed EGFP were used [32,33]. BHK-21 cells were infected with the two viruses, respectively, as described above, and then incubated at different temperatures. The above assays have revealed that RGV and ADRV can replicate in BHK-21 at 28 °C, so the temperatures 31, 34, and 37 °C with an interval of 3 °C were first tested. Then, a middle temperature (32.5 °C) between 31 and 34 °C was tested. The cells were observed under a fluorescence microscopy (OLYMPUS IX73, Tokyo, Japan) at 48 hpi.

In RT-PCR detection of the virus infection, RGV, ADRV, or SMRV was used to infect BHK-21 cells, which was incubated at 37 °C. The cells were collected at different time points for RT-PCR.

### 2.8. Detection of Virus Gene Expression by RT-qPCR and RT-PCR

RNA was extracted from the collected cells described above using a FastPure Cell/Tissue Total RNA Isolation Kit (Vazyme, Nanjing, China), and first-strand cDNA synthesis was performed with HiScript III RT SuperMix for qPCR (+gDNA wiper) (Vazyme, Nanjing, China). RT-qPCR was conducted on a Quantagene q225MX Real-Time PCR Detection System (Kubo, Beijing, China). Each RT-qPCR mixture contained 5 μL of SYBR Premix (2×), 0.5 μL of forward and reverse primers (for each primer), 1 μL of cDNA, and 3 μL of ultrapure water. The RT-qPCR conditions were as follows: 95 °C for 5 min; 40 cycles of 95 °C for 15 s, 60 °C for 40 s; and a melt curve analysis from 60 to 95 °C. The MCP gene of RGV and ADRV, N gene of SMRV and PORV, and VP6 gene of GCRV were detected. The β-actin gene was used as internal control. The mRNA relative expression ratios of the treated group versus that of the control group were calculated by the 2^-ΔΔCT^ method.

For RT-PCR, three genes (*ICP18*, *dUTPase*, and *MCP*) were detected in RGV and ADRV infected cells and *N* gene was detected in SMRV infected cells, which were performed as described previously [34]. The genes and primers used in the study were collected in Supplementary Table S1.

## 3. Results

### 3.1. Effect of UV Radiation Treatment

**Figure 1:**
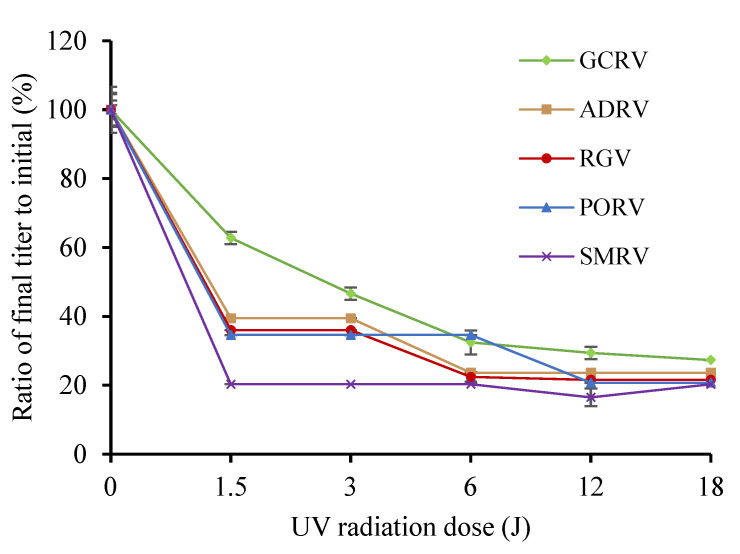
Effect of UV dose on titers of RGV, ADRV, PORV, SMRV, and GCRV. Each point in the figure showed the ratio of the titer after UV radiation treatment to initial titer for each virus.

Titers of five viruses were measured after a wavelength of 254 nm UV treatment. As shown in Figure 1, with the increase in UV dose—0, 1.5, 3, 6, 12, 18 J (0, 5, 10, 20, 40, 60 min)—titers of viruses decreased strikingly. After the UV treatment with a radiation energy of 1.5 J, the viruses with titers from high to low were GCRV, RGV, ADRV, PORV and SMRV. Their TCID_50_ were 10^3.4^, 10^2.5^, 10^2.5^, 10^2.5^, and 10^1.5^, respectively, which have a decay rate of 37%, 54%, 61%, 65%, and 80%, respectively, compared to their initial titer (Figure 1). Then, titers of GCRV, PORV, ADRV, and RGV decreased slowly from the time point to the end of the test, while that of SMRV showed an undetectable decrease.

When the treatment energy increased to 18 J, the titers of the five viruses decreased to about 10^1.5^ TCID_50_, with a decay rate of 73%, 78%, 76%, 79%, and 80% for each virus above. The results indicated that UV treatment highly influenced the virus activities, but the nonenveloped dsRNA virus GCRV was most resistant to the UV treatment, while SMRV was most sensitive.

### 3.2. Effect of Incubation at Different Temperatures

**Figure 2:**
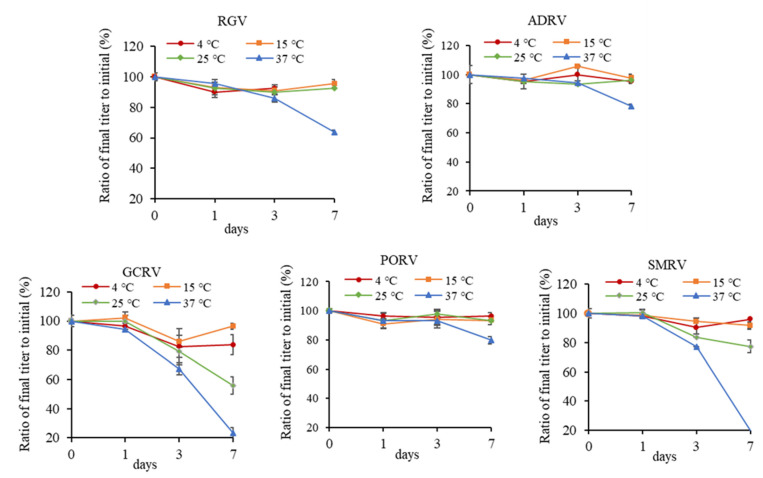
Effect of different temperatures (4, 15, 25, and 37 °C) on titers of five aquatic animal viruses. Each point in the figure showed the ratio of the titer after incubation to initial titer for each virus.

Titers of the five viruses were measured after incubating at 4, 15, 25, and 37 °C for 1, 3, and 7 days, respectively. As shown in Figure 2, under most circumstances, no major effects on titers were measured after incubating at 4, 15, and 25 °C, except that SMRV and GCRV showed a reduction after incubating at 25 °C for 7 days. At this moment, the titers decreasing from low to high were ADRV, RGV, PORV, SMRV, and GCRV. TCID_50_ were 10^6.7^, 10^7^, 10^6.7^, 10^6.0^, and 10^2.7^, respectively.

After incubating at 37 °C for 7 days, titers of all of the five viruses were obviously reduced. The titers decreasing from low to high were PORV, ADRV, RGV, GCRV, and SMRV, with a TCID_50_ of 10^5.9^, 10^5.4^, 10^4.6^, 10^1.1^, and 10^1.5^, respectively. The decay rates were 20%, 22%, 36%, 77%, and 81%, respectively. It indicated that SMRV and GCRV were more sensitive to thermal stress, while other viruses could be stable for longer time.

### 3.3. Effect of pH Stress

**Figure 3:**
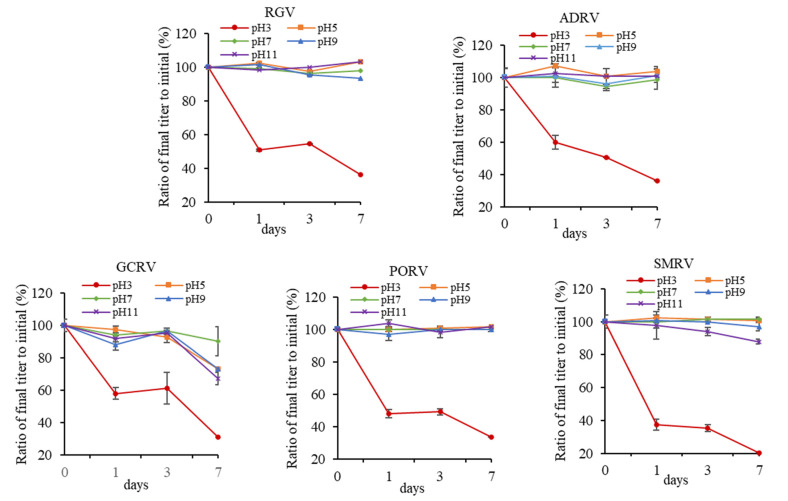
Effect of different pH (3, 5, 7, 9, and 11) on titers of five aquatic animal viruses. Each point in the figure showed the ratio of the titer after treatment to initial titer for each virus.

Titers of the five viruses were measured after treatment with different pH for 1, 3, and 7 days, respectively. As shown in Figure 3, no obvious effects on titers were obtained after treated by pH 5~11 for 7 days, except for GCRV. After pH 5, 9, and 11 treatments for 7 days, TCID_50_ of GCRV were 10^3.5^, 10^3.5^, and 10^3.2^, with a decay rate of 33%, 33%, and 39%, respectively. However, titers of the viruses all decreased strikingly at pH 3. After being treated for 1 day, the titers decreasing from low to high were ADRV, RGV, GCRV, PORV, and SMRV, with a TCID_50_ of 10^4.2^, 10^3.9^, 10^2.8^, 10^3.6^, and 10^2.8^, respectively, and the decay rates were 40%, 46%, 47%, 52%, and 62%, respectively.

After treatment with pH 3 for 7 days, the titers decreasing from low to high were, respectively, ADRV, RGV, PORV, GCRV, and SMRV. TCID_50_ were 10^2.5^, 10^2.6^, 10^2.5^, 10^1.5^, and 10^1.5^, with a decay rate of 64%, 64%, 66%, 71%, and 80%, respectively. It indicated that GCRV was sensitive to a wide range of pH, even nearly the neutral pH, and all of the five viruses were sensitive to strong acidic pH of 3, especially SMRV.

### 3.4. Effect of Drying Stress

**Figure 4:**
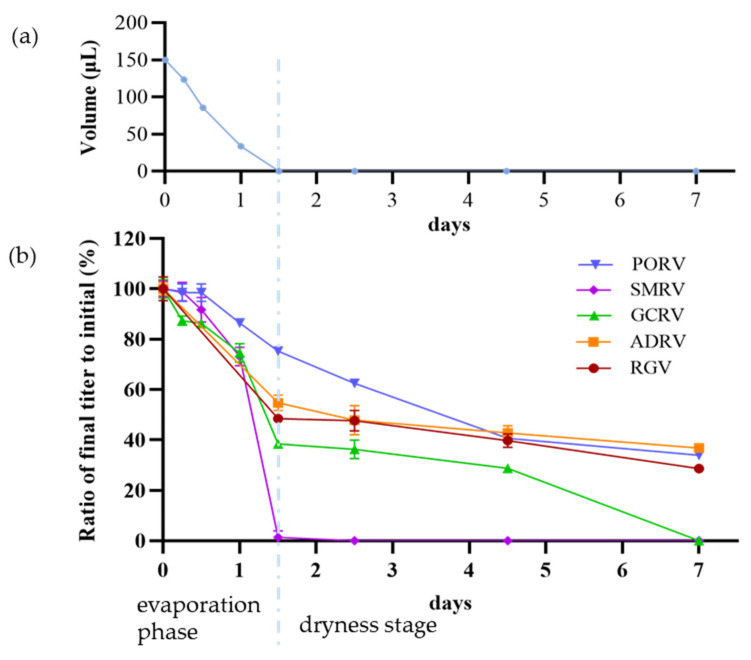
Drying process and effects of drying stress on titers of five aquatic animal viruses. (**a**) Drying process was divided into two phases: an evaporation phase (0~1.5 d, the front part) during which the volumes of virus suspensions decreased from 150 μL to 0 μL, and a dryness stage (1.5~7 d, the latter part), in which the virus suspensions have completely dried. (**b**) The decrease in virus titers in drying process. Each point showed the ratio of the titer after treatment to initial titer for each virus.

In the drying assays (25 °C and 70~80% RH), the total process could be divided into two stages, the evaporation phase (0~1.5d, in which the volumes of virus suspensions decreased from 150 μL to 0) and the quasi-equilibrium phase (1.5~7 d, in which the virus suspensions have completely dried), as shown in Figure 4a. Titers of the five viruses were measured after drying treatment for 0, 6, 12, 24, 36, 60, 108, and 168 h, respectively, as shown in Figure 4b. With the prolonging of drying time, titers of viruses decreased fast at the end of the evaporation phase (1.5 d). At this moment, the titers decreasing from low to high were PORV, ADRV, RGV, GCRV, and SMRV. TCID_50_ were 10^5.6^, 10^3.6^, 10^3.4^, 10^2.0^, and 10^0.1^, with a decay rate of 25%, 45%, 52%, 62%, and 99%, respectively. With the further extension of drying time (dryness stage), the reduction in titers of viruses slowed down. Finally, the titers decreasing from low to high were, respectively, ADRV, PORV, RGV, GCRV, and SMRV. TCID_50_ were 10^2.4^, 10^2.5^, 10^2^, 10^0^, and 10^0^, with a decay rate of 63%, 66%, 71%, 100%, and 100%, respectively. It indicated that SMRV was the most sensitive virus to drying stress, losing titers soon after completely dried, which suggested that drying could be an effective strategy to inactivate SMRV.

### 3.5. Sensitivity of the Five Viruses to BHK-21 Cells

**Figure 5:**
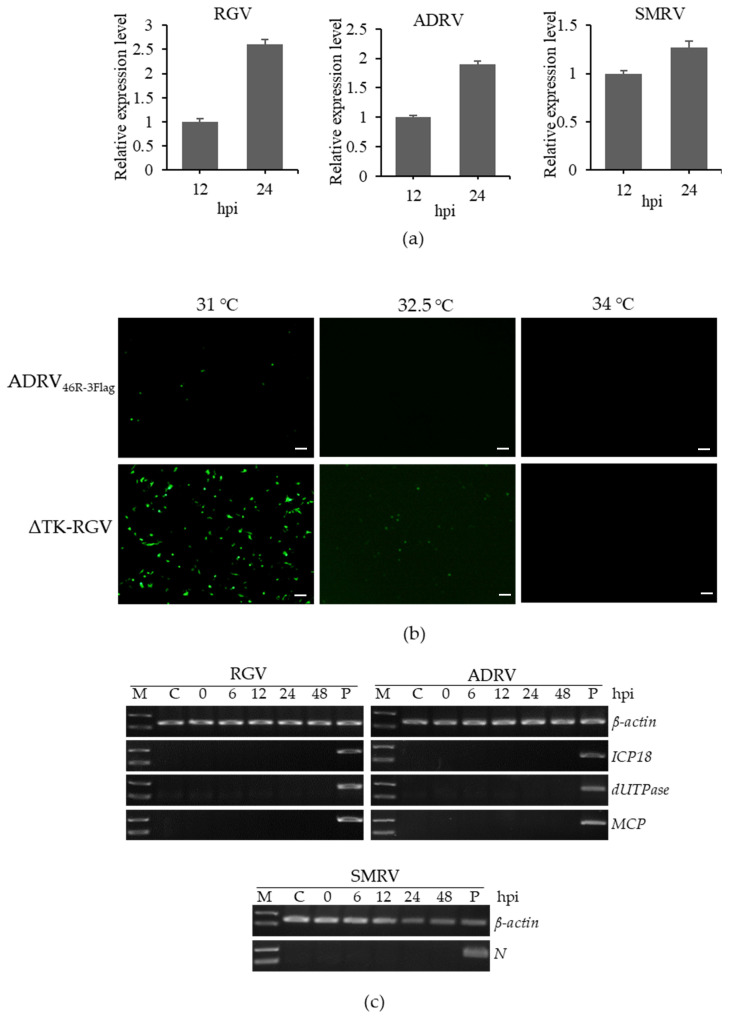
Detection of the infectivity of the aquatic animal viruses in BHK-21 cell. (**a**) Gene expression of the five viruses detected by RT-qPCR. The three viruses, RGV, ADRV, and SMRV, that had gene expression in the cells were shown. (**b**) Fluorescence microscopy observation of the cells infected by ADRV_46R-3Flag_ and ΔTK-RGV at different temperatures. The green fluorescence was not observed at the temperatures more than 32.5 °C for ADRV_46R-3Flag_ and more than 34 °C for ΔTK-RGV. Bar = 200 μm. (**c**) RT-PCR detection of the gene expression of RGV, ADRV, and SMRV at 37 °C in BHK-21 cells. Expression of *ICP18*, *dUTPase*, and *MCP* gene of RGV and ADRV, and *N* gene of SMRV cannot be detected. *ICP18*, *dUTPase*, and *MCP* gene are representative genes of different transcription stages of RGV and ADRV. P, positive control. The detection of β-actin was used as internal control.

Replication of the viruses was determined by RT-qPCR detection of their gene expression in virus infected BHK-21 cells. For the five viruses, the expression of the *MCP* gene of RGV and ADRV and *N* gene of SMRV was significantly increased from 12 to 24 hpi (Figure 5a), while the expression of the GCRV and PORV genes was not detected, indicating the three viruses, RGV, ADRV, and SMRV, can infect BHK-21 cells.

RGV and ADRV are ranaviruses which can infect interspecies in aquatic animals. We analyzed the effect of culture temperature on their ability to infect BHK-21 cells. BHK-21 cells were infected with recombinant ADRV or RGV that expressed EGFP and incubated at different temperatures. As shown in Figure 5b, the infection of the two viruses in BHK-21 cells both decreased rapidly with the increase in the culture temperature. Under a temperature of less than 31 °C, the expression of EGFP can be observed in ADRV_46R-3Flag_ infected BHK-21 cells, but it cannot be observed when temperature was more than 32.5 °C. For ΔTK-RGV infection, expression of EGFP cannot be observed in cells culture at temperatures more than 34 °C.

To confirm the virus infectivity in BHK-21 cells at 37 °C, gene expressions of the three viruses, RGV, ADRV, and SMRV, were detected by RT-PCR when incubated at 37 °C. As shown in Figure 5c, expression of the β-actin gene was detected in all of the samples, but none of the genes’ expression (*ICP18*, *dUTPase*, and *MCP*, which are representative genes of different transcription stages of RGV and ADRV, and *N* for SMRV) can be detected in different time points after infection, indicating the three viruses cannot be replicated in BHK-21 cells at 37 °C.

## 4. Discussion

As a large group of microorganisms, viruses respond to various physicochemical factors in the environment. In this study, the effects of four environmental factors on five aquatic animal viruses were compared, and the virus infectivity on mammalian cells was analyzed. Many attractive results were observed, such as two rhabdoviruses SMRV and PORV, which showed divergent resistance to the same environmental factor; GCRV presented sensitivity to pH and resistance to UV; the enveloped dsDNA virus RGV and ADRV performed high tolerance to the tested factors; and some viruses can infect BHK-21 cell but they are limited by temperature, as shown in Table 1.

PORV and SMRV are both in the family *Rhabdoviridae*. In this study, it was observed that SMRV was the most sensitive virus to UV, thermal, strong acidic pH, and drying stresses, which was significantly different to PORV, especially when encountering thermal and drying stresses. As previously reported, they, respectively, belong to genus *Novirhabdovirus* and *Vesiculovirus.* There is an NV gene in the former genome, while it does not exist in the latter. The gene has been studied in other fish rhabdoviruses, which identified its important role in supporting virus replication [35,36]. However, whether the product of NV gene weakens the destruction of physicochemical stresses remains an interesting question to us, which needs further study. In addition, it should be noted that the propagation temperature for PORV is 15 °C but 25 °C for SMRV, which may lead to some differences in proteins between the two viruses.

GCRV was sensitive to pH, even nearly the neutral pH. There were no obvious differences among GCRV and other viruses in the face of pH 3. However, its TCID_50_ significantly decreased in the weak acid or alkaline environment compared to other viruses. As previously reported, the nonenveloped reoviruses have two pH sensing proteins in capsid proteins, one of which can detach from the capsid and the other of which can undergo structural changes at low and high pH, thus affecting infection efficiency [37,38]. GCRV is also a reovirus, and its S10 segment encodes a VP7 capsid protein which also contains a possible pH sensor domain thus leading to its sensitive to pH [18,39], which is worthy of concern. On the other hand, GCRV was most resistant to UV radiation, and this is consistent with previously reported results of the dsRNA virus genome was more resistant to UV_254_ exposure than the dsDNA virus and ssRNA virus genomes [40].

Our results about RGV and ADRV showed that they both were relatively resistant to various tested environmental factors in this study. There have been several studies involved in environmental resistance of ranaviruses in laboratories or natural conditions [41,42,43]. They survive at desiccation, high temperatures, and so on [44]. This high resistance might provide a strong survival ability for these kind of viruses and also be consistent with the wide distribution of ranaviruses in the world, and even their ability to transmit across species [45,46].

On the whole, the tested viruses were all more sensitive to high temperature and low pH than low temperature and high pH, which could be related to the low stability or low enzymatic activity of the viral proteins during these situations. Although the exact mechanisms need future exploration, the unfavorable environmental factors to the viruses could be considered in disease control of aquatic animals.

Cross-species infection has been reported in some aquatic animal viruses, such as ranaviruses in the family *Iridoviridae* [45]. In the present study, three of the five viruses showed the infectivity to the BHK-21 cells at a low temperature, which indicated that the intracellular environment of mammalian cells can support the replication of some aquatic animal viruses. However, these viruses cannot replicate at 37 °C, which is the normal physiological temperature for most mammals, including humans. We also tested the infectivity of the two ranaviruses in a cultured human cell line, which showed similar results to those in BHK-21. Aquatic viruses have adapted to the aquatic environment, which has a low temperature and is a place whereby most of the fish cell lines are cultured under 15–28 °C. So, components of aquatic virus such as enzymes have adapted to the low temperature, which could lose activity at 37 °C and inactivate the virus.

## 5. Conclusions

The infectivity of five aquatic animal viruses under four kinds of environmental factors were tested and compared in fish cell, which showed that the tolerance of these viruses to the factors might be related to various aspects, such as the enveloped dsDNA viruses RGV and ADRV are relatively stable, but the ssRNA rhabdovirus SMRV is sensitive to UV (dose 1.5J), thermal (37 °C), and drying stresses. In addition, the aquatic animal viruses cannot replicate in mammalian cells under a normal physiological temperature, indicating aquatic animal viruses cannot propagate in mammals.

## Figures and Tables

**Table 1:** TCID_50_ (decay rates) of viruses under various environmental factors in fish cell and the infectivity in mammalian cell.

Viruses		RGV	ADRV	GCRV	PORV	SMRV
Family		*Iridoviridae*	*Iridoviridae*	*Reoviridae*	*Rhabdoviridae*	*Rhabdoviridae*
Genome		dsDNA	dsDNA	dsRNA	ssRNA	ssRNA
Genome size		105.8 kb	106.7 kb	25 kb	11.2 kt	11.5 kt
ORFs		106	101	11	6	5
Diameter (nm)		150	150~160	55~80	60 × 200	40~60 × 110~150
Enveloped		yes	yes	no	yes	yes
Cell type	Environmental factor	TCID_50_ (decay rate compared to primary TCID_50_)				
Fish cell (SCSC)	UV dose: 1.5 J	10^2.5^ (54%)	10^2.5^ (61%)	10^3.4^ (37%)	10^2.5^ (65%)	10^1.5^ (80%)
	UV dose: 18 J	10^1.5^ (78%)	10^1.5^ (76%)	10^1.5^ (73%)	10^1.5^ (79%)	10^1.5^ (80%)
	25 °C, 7 d	10^6.7^ (7%)	10^6.7^ (4%)	10^2.7^ (44%)	10^7.0^ (5%)	10^6.0^ (23%)
	37 °C, 7 d	10^4.6^ (36%)	10^5.4^ (22%)	10^1.1^ (77%)	10^5.9^ (20%)	10^1.5^ (81%)
	pH = 3, 1 d	10^3.9^ (46%)	10^4.2^ (40%)	10^2.8^ (47%)	10^3.6^ (52%)	10^2.8^ (62%)
	pH = 3, 7 d	10^2.6^ (64%)	10^2.5^ (64%)	10^1.5^ (71%)	10^2.5^ (66%)	10^1.5^ (80%)
	Evaporation, 1.5 d	10^3.4^ (52%)	10^3.6^ (45%)	10^2.0^ (62%)	10^5.6^ (25%)	10^0.1^ (99%)
	Dryness, 7 d	10^2.0^ (71%)	10^2.4^ (63%)	10^0^ (100%)	10^2.5^ (66%)	10^0.0^ (100%)
Mammalian cell (BHK-21)	Infect at 28 °C	+	+	-	−	+
	Infect at 34 °C	−	−	−	−	
	Infect at 37 °C	−	−	−	−	−

Decay rate = (lg*iTCID_50_*-lg*fTCID_50_*)/lg*iTCID50*, in which the *iTCID_50_* indicate the initial titer of each virus, and *fTCID_50_* indicate the final titer obtained after different treatment. “+” and “−” indicate “infected” and “uninfected”, respectively.

## Data Availability

Not applicable.

## Supplementary Materials

The following supporting information can be downloaded at: https://www.mdpi.com/article/10.3390/v14112546/s1, Table S1: Primers used in the study.
