# Supplementary material for: Environmental Factors and Their Threshold Affecting the Survival of Five Aquatic Animal Viruses in Different Animal Cells
Source: Viruses. 2022 Nov 17;14(11):2546. doi: 10.3390/v14112546 (PMC9696523; doi:10.3390/v14112546)
Supplement: Supplementary file 1 [file viruses-14-02546-s001.zip › viruses-2030726-supplementary.pdf]

Table S1. Primers used in the study.

gene	Forward primer (5′-3′)	Reverse primer (5′-3′)
ICP18	ACGACCTGGGAACCTTTGTG	GTTGTCGTACAGGCATTCGC
dUTPase	AACCTCGGCGTCATACTGTT	TCACCCCTGTCGGTAGAGTC
MCP	CACCTCCATCCCAGTCAGCA	AATCCCATCGAGCCGTTCA
SMRV-*N*	GAGATGGTCAACTGGGGGTG	ACCGGAGCGTCTTTCCATTT
GCRV-*VP6*	ACAACGGCTGCTTTGATGGC	TCCGTTGCAAGTGCGAGAGCG
PORV-*N*	TCACCGCCGACAAAGCA	GTCCGATCATGGTCATTCCTG
β-actin	CCAAGGCCAACCGTGAAAAG	TCCGGAGTCCATCACAATGC
